# Association Between Screen Time, Fast Foods, Sugar-Sweetened Beverages and Depressive Symptoms in Chinese Adolescents

**DOI:** 10.3389/fpsyt.2020.00458

**Published:** 2020-05-26

**Authors:** Honglv Xu, Jichang Guo, Yuhui Wan, Shichen Zhang, Rong Yang, Huiqiong Xu, Peng Ding, Fangbiao Tao

**Affiliations:** ^1^ Department of Maternal, Child and Adolescent Health, School of Public Health, Anhui Medical University, Hefei, China; ^2^ MOE Key Laboratory of Population Health Across Life Cycle Anhui Medical University, Hefei, China; ^3^ School of Education Science, Yulin Normal University, Yulin, China

**Keywords:** depressive symptoms, screen time, dietary behavior, association, adolescent

## Abstract

**Objective:**

Although previous studies have shown that screen time (ST), fast foods (FFs) and sugar-sweetened beverages (SSBs) consumption are associated with depressive symptoms in adolescents, research on these associations in Chinese adolescents is scarce. This study aimed to examine the association between ST, FFs, SSBs and depressive symptoms in Chinese adolescents, and explore the mediating effects of FFs and SSBs in the association between ST and depressive symptoms.

**Methods:**

This school-based nationwide survey was carried out among 14,500 students in four provinces of China. *The Children’s Depression Inventory* was used to assess the participants’ depressive symptoms. ST, FFs and SSBs consumption was measured by a self-reported questionnaire. The Bayesian multiple mediation model was used to analyze the mediation effect.

**Results:**

ST, FFs and SSBs, were more likely to be associated with depressive symptoms, and *ORs* (*95%CI*) was 1.075 (1.036–1.116), 1.062 (1.046–1.078) and 1.140 (1.115–1.166), after we adjusted for sociodemographic variables. Additionally, in Bayesian multiple mediation model, direct effect, mediating effect, total effect, the ratio of mediating effect to total effect was 0.125, 0.034, 0.159, and 0.214, respectively. All path coefficients of the three mediation paths are statistically significant (*p* < 0.05).

**Conclusions:**

Our study demonstrates that ST, FFs and SSBs consumption are associated with depressive symptoms in Chinese adolescents. It is likely that FFs and SSBs partially mediate the association between ST and depressive symptoms by chain-mediating effects.

## Introduction

The World Health Organization previously reported that more than 300 million people worldwide suffer from depression (1). It was hypothesized that depression would be the main cause of the global burden of disease by 2030 (2). Worldwide prevalence rate suggests that there is approximately 11.00% of depression among adolescent (3), and the rate increases with age (4). A study based on the structured diagnostic interview of DSM-IV-TR reports that the prevalence of depression in German adolescent was 27.3% (5). The positive rates of depressive symptoms among adolescents in China, Japan and France were 44.3, 22.0 and 12.6%, respectively (6–8). Moreover, the positive rate of depressive symptoms in adolescents in developing countries may be higher, compared with developed countries. For instance, a study involving three countries shows that the positive rate of depression among adolescents in India is higher than that in Australians and Americans (9). Notably, severe depression can contribute to suicide, and suicide is the second cause of death in people aged 15–29 (1). Depression has been identified as one of the important factors influencing the health of adolescents (10). Thus, the American Academy of Pediatrics recommended that adolescents should be screened for depressive symptoms at least once a year (10). Likewise, the World Health Assembly calls for the prevention and treatment of mental illnesses such as depression at the national level (1).

Approximately 50.0% of children and adolescents spend more than 2 h a day on the screen (11). For example, about 45.0% of adolescents aged 15–19 of Moroccan watch TV for more than 2 h per day, and 38.0% of them use computers at the same time (12). Recent evidence suggests a link between ST and adolescent depression (4, 13), and clear dose–response relationships were observed (14). A Chinese study indicates that watching TV on weekdays for more than 2 h is associated with an increased risk of depression for boys (*OR* = 1.33, 95%CI: 1.02–1.73) and girls (*OR* = 1.62, 95%CI: 1.19–2.21) (13). Besides, video games (*β* = 0.13, *p* < 0.001) and computer use (*β* = 0.17, *p* < 0.001) for a long time are associated with more severe depressive symptoms in Canadian adolescents (15).

There are growing interests in the association between dietary behavior and depression (16, 17). Several considerable researches suggest that consumption of highly processed foods (e.g., FFs, sweet foods, fried foods, processed meat, etc.) are associated with depression (18, 19), but not vice versa, healthy foods (e.g., fruits, vegetables, nuts, whole grains, etc.) are negatively correlated with depression (20–22). Of interest, adolescents consume more FFs and SSBs in the context of the globalization (23, 24). Approximately 45.4 and 19.6% of adolescent aged 15–19 consume SSBs and FFs more than three times a week (12). Also, the previous study suggests that frequent consumption of FFs and SSBs may be more strongly associated with depression in adolescents (7, 25–27). Notably, a nationwide survey of 65,212 adolescents in South Korea shows that unhealthy dietary consumption was positively correlated with perceived stress and depressive symptoms (17).

Although potential associations were described above, there are also some inconsistent research findings. For instance, a study has found no explicitly association between TV time and adolescent depressive symptoms (15). And more studies have reported Western dietary patterns are not associated with depressive symptoms in adolescents (28, 29), and healthy dietary consumption cannot predict adult depression (10, 30). Furthermore, to our knowledge, previous studies have rarely analyzed the association between ST, FFs, SSBs and depressive symptoms, few have concerned about the intermediary mechanism of this association. Based on the results of the reviews mentioned above, we hypothesized that there might be mediating effects of FFs and SSBs in the association between ST and depressive symptoms in adolescents. In this study, we used data of cross-sectional investigation from China to analyze the mediation model.

## Materials and Methods

### Participants

This school-based nationwide questionnaire survey was carried out among middle school students aged 10–20 years (14.9 ± 1.8) from 32 middle schools in four provinces of China (Shenzhen, Guangdong Province; Zhengzhou, Henan Province; Nanchang, Jiangxi Province; Guiyang, Guizhou Province) from November 2017 to January 2018. Some 15,486 students were surveyed, and 871 (5.6%) adolescents rejected to participate in the survey. A total of 14,615 (94.4%) questionnaires were completed, and 14,500 (99.2%) questionnaires were analyzed, including 7,347 (50.7%) boys and 7,153 (49.3%) girls. Moreover, there were 6,881 (47.5%) rural residents and 7,619 (52.5%) urban residents. The distribution of other sociodemographic variables is shown in **Table 1**.

**Table 1:** The positive rate of depressive symptoms in Chinese adolescents of stratified by gender (%).

Variables		Male (n = 7,347)				Female (n = 7,153)			
		N	Depressive symptoms (%)	χ^2^	*p*	N	Depressive symptoms (%)	χ^2^	*p*
Age(year)	10–15	4,230	1,233 (29.1)	0.447	0.504	4,557	1,250 (27.4)	10.127	0.001
	16–20	3,117	931 (29.9)			2,596	804 (31.0)		
Grade	1	1,133	270 (23.8)	26.294	<0.001	1,291	288 (22.3)	25.802	<0.001
	2	1,210	301 (24.9)			1,206	310 (25.7)		
	3	1,194	336 (28.1)			1,213	312 (25.7)		
	4	1,189	372 (31.3)			1,244	377 (30.3)		
	5	1,220	374 (30.7)			1,198	347 (29.0)		
	6	1,401	388 (27.7)			1,001	277 (27.7)		
Residence	Rural	3,443	1,034 (30.0)	16.377	<0.001	3,438	948 (27.6)	2.49	0.115
	City	3,904	1,007 (25.8)			3,715	963 (25.9)		
The only child in the family	Yes	2,767	762 (27.5)	0.129	0.720	1,902	477 (25.1)	3.547	0.060
	No	4,580	1,279 (27.9)			5,251	1,434 (27.3)		
Boarding school	Yes	3,506	911 (26.0)	10.782	0.001	3,324	883 (26.6)	0.073	0.787
	No	3,841	1,130 (29.4)			3,829	1,028 (26.8)		
Father’s education level^★^	Illiteracy	281	111 (39.5)	77.578	<0.001	283	104 (36.7)	43.422	<0.001
	Elementary school	663	226 (34.1)			788	216 (27.4)		
	Secondary school	2,333	668 (28.6)			2,373	674 (28.4)		
	High school	2,146	556 (25.9)			1,974	481 (24.4)		
	The university	1,832	433 (23.6)			1,647	397 (24.1)		
Mother’s education level^•^	Illiteracy	561	193 (34.4)	67.46	<0.001	608	210 (34.5)	50.5	<0.001
	Elementary school	996	303 (30.4)			1,004	294 (29.3)		
	Secondary school	2,266	628 (27.7)			2,398	637 (26.6)		
	High school	1,980	534 (27.0)			1,806	438 (24.3)		
	The university	1,461	336 (23.0)			1,274	301 (23.6)		
Self-perceived socioeconomic status	Worse	832	310 (37.3)			698	294 (42.1)	179.643	<0.001
	Poor	334	165 (49.4)	148.276	<0.001	175	92 (52.6)		
	Medium	4,821	1,232 (25.6)			5,189	1,298 (25.0)		
	Good	241	83 (34.4)			172	51 (29.7)		
	Better	1,119	251 (22.4)			919	176 (19.2)		
The number of close friend	0	291	182 (62.5)	319.523	<0.001	146	88 (60.3)	254.791	<0.001
	1–2	1,355	524 (38.7)			1,744	650 (37.3)		
	3–5	2,846	720 (25.3)			3,305	804 (24.3)		
	≥6	2,855	615 (21.5)			1,958	369 (18.8)		

★ There are 180 adolescents（92 male students, 88 female students）without fathers.

• There are 146 adolescents (83 male students, 63 female students) without mothers.

Values in parentheses represent the positive rate of depressive symptoms.

### Procedures

Participants were selected by cluster and the stratiﬁed multistage sampling method. As well, research areas were selected according to China’s geographical distribution, economic development level and cooperation with our research team. Next, four urban and four rural middle schools in each area were randomly sampled, and 400 students were surveyed of each school, including all grades. Students were gathered in the classroom to complete the questionnaire in about 30 minutes. Notably, teachers maintain order in the classroom to ensure that students complete the questionnaire independently and do not discuss the content of investigation each other. The principles of anonymity, confidentiality and voluntariness are strictly followed in our study. Participants and their guardians provide informed consent, and students can withdraw from the study at any time. This study was approved by the Ethics Committee of Anhui Medical University (batch number: 20140087). The detailed survey information can be found in our previous article (31).

### Sociodemographic Variables

Gender, age, grade, residence, father’s education level, mother’s education level, the only child in the family or not, the number of close friends, boarding school, self-perceived socioeconomic status were included as covariates.

### Depressive Symptoms Assessment

*The Children’s Depression Inventory* (CDI) was used to assess depressive symptoms in adolescents (32). CDI is a self-report internationally popular scale consisting of 27 items for assessing depressive symptoms in children and adolescents (33). Each item consists of three sentences, such as “I feel sad at times”, “I often feel sad” and “I always feel sad”, which is assigned a score from 0 to 2. The total score ranges from 0 to 54, with a higher score being attributed to the most depressive state. The total score of 19 was determined as a threshold for assessing depressive symptoms according to the scale norm. Namely, a total score of 19 or more was assessed as a positive depressive symptom. In contrast, a total score of less than 19 was assessed as a negative depressive symptom. Additionally, its widespread use confirmed that CDI has good reliability and validity, and the Cronbach α was 0.87 in another study (34).

### Screen Time Assessment

ST was measured by two questions: asking students to report the amount of time (hour) spent watching television, using computer and smartphone during weekdays (from Monday to Friday) and on week-ends (from Saturday to Sunday). For instance, how many hours do you spend watching television, using a computer and smartphone during weekdays in the latest week? How many hours do you spend watching television, using computer and smartphone on week-ends in the latest week? There are seven options for each question as follows: 1, 0 h; 2, < 1 h; 3, 1–2 h; 4, 2–3 h; 5, 3–4 h; 6, 4–5 h; 7, ≥5 h, which is assigned a score from 0 to 6. For the analysis, the weekly ST was calculated by adding the scores of the two items together, with the higher score being attributed to the longer ST.

### FFs and SSBs Consumption Assessment

Five kinds of FFs and five kinds of SSBs consumption were investigated in this study. FFs include Western-style FFs (e.g., McDonald’s, etc.), Chinese FFs (e.g., Shaxian snacks, etc.), takeaway FFs (e.g., Meituan takeaways, etc.), foods brought from the school cafeteria and those brought from off-campus restaurant packed in a disposable fast food box or plastic bags. Also, SSBs include carbonated drinks (e.g., Sprite, etc.), fruit and vegetable juice drinks (e.g., fruit orange, etc.), tea drinks (e.g., iced black tea, etc.), energy drinks (e.g., Red Bull, etc.), and dairy beverages (e.g., milk tea, etc.). The self-reported food frequency questionnaire (FFQ) was used to assess the consumption of FFs and SSBs in the last week. For example, how many times have you eaten Western-style FFs in the last week (e.g., McDonald’s, etc.)? How many bottles of energy drinks do you drink every day in the last week (e.g., Red Bull, etc.)? Four options per question (1, never; 2, 1–2 times/1 bottle; 3, 3–4 times/2–3 bottles; 4, ≥5 times/≥4 bottles) were assigned a score from 0 to 3. The total score for FFs and SSBs was calculated by the scores of five kinds of FFs and five kinds of SSBs, respectively, with the higher score being attributed to the higher frequency consumption. In the present survey, the Cronbach α of FFQ was 0.77.

### Statistical Analyses

The database was created by EpiData 3.0. Statistical analyses were performed with Mplus (Mplus Version 7.4) and SPSS 23.0 (SPSS Inc, Chicago, IL). Measurement data is expressed as (mean ± SD). Analytical methods included descriptive statistical analysis, the chi-squared test, common method biases test, logistic regression model, and Bayesian mediation model. Normality test, common method biases test and logistic regression analysis were performed in SPSS software. Participants’ depressive symptom scores were converted into binary classification variables based on a positively defined cut-off value (≥19). Then, the chi-square test was used to compare the positive rates of depressive symptoms among adolescents with different demographic characteristics. The common method biases test was performed by Harman single factor test, which is an exploratory factor analysis with no factor rotation and no specified the number of extraction factors. Additionally, we used logistic regression model to analyze the association of ST, FFs and SSBs with depressive symptoms. ST, FFs and SSBs were continuous variables, and depressive symptoms were binary variables in a model. ST, FFs and SSBs were independent variables, and depressive symptoms were dependent variables. Two models were constructed: Model 1 unadjusted variables, and Model 2 adjusted demographic variables. We calculated the odds ratios (*OR*s) and 95% confidence intervals (95% *CIs*) in the logistic regression model.

The mediation effect analysis was performed by Bayesian mediation model, and the Markov chain Monte Carlo (MCMC) method was used for parameter estimation in Mplus software (35, 36). Model convergence was tested by Bayesian posterior parameter distributions plots, trace plots and Bayesian autocorrelation plots. Meanwhile, Bayesian posterior predictive checking using the chi-square test, and the *P* value was obtained by the chi-square value likelihood ratio test of the hypothesis model and the free estimation model. Besides, the model was evaluated by scatter plots and distribution plots of Bayesian posterior predictive checking (PPC) (37, 38). Mediation model indicators (e.g., direct effect, mediating effect, total effect, the ratio of mediating effect to total effect, the ratio of mediating effect and direct effect, mediating effect through mediating variables) were calculated to determine the critical mediation variables and paths. Particularly, we used structural equation model to analyze the mediating effects as a sensitivity analysis (39). Parameter estimation was performed with maximum likelihood, and path coefficient test was performed with the Bootstrap method (40, 41). The significant level was α = 0.05.

## Results

### Sociodemographic Variables and Positive Rate of Depressive Symptoms in Adolescents

**Table 1** shows the positive rates of depressive symptoms in adolescents with different sociodemographic variables. 27.3% (3,952/14,500) adolescents were diagnosed with positive depressive symptoms in the total study population. The positive rates of depressive symptoms were statistically different in sociodemographic variables of stratified by gender (*P* < 0.05) except different ages and whether the only child in the family of male (*P* > 0.05), and different residences, whether boarding school and whether the only child in the family of female (*P* > 0.05).

### Common Method Biases Test

Eight common factors with characteristic root greater than one were extracted in exploratory factor analysis, and 15.26% (< 40%) variance was explained by the first common factor (42). The results show that the common method biases were not significant, and the correlation among variables is credible in our study.

### Correlation Analysis

**Table 2** shows the significant association between the positive rate of depressive symptoms, ST, FFs and SSBs in the logistic regression model (*P* < 0.01). More significant associations were observed after adjusting for sociodemographic variables (*p* < 0.01), and the *OR*s values of the ST, FFs and SSBs were 1.075 (95%CI:1.036–1.116), 1.062 (95%CI:1.046–1.078), and 1.140 (95%CI:1.115–1.166), respectively.

**Table 2:** Association between the positive rate of depressive symptoms, ST, FFs and SSBs.

Model^▲^	Variables	B	S.E.	Wald	*P*	*OR*	95% C.I.
Model 1	ST score	0.071	0.007	119.003	<0.001	1.074	1.060–1.088
	FFs score	0.060	0.007	66.901	<0.001	1.062	1.046–1.077
	SSBs score	0.112	0.010	122.001	<0.001	1.119	1.097–1.141
Model 2	ST score	0.072	0.019	14.436	<0.001	1.075	1.036–1.116
	FFs score	0.060	0.008	58.564	<0.001	1.062	1.046–1.078
	SSBs score	0.131	0.011	137.403	<0.001	1.140	1.115–1.166

▲ Model 1, unadjusted variable. Model 2, adjusted for sociodemographic variables.

### Mediating Effect Analysis

**Table 3** shows the mediating effects of FFs and SSBs in the association between ST and depressive symptoms in adolescents. Notably, the direct effect of ST on depressive symptoms was 0.125; the total mediating effect is 0.034; the total effect was 0.159. The ratio of direct effect to total effect, mediating effect to total effect and mediating effect to direct effect was 0.786, 0.214 and 0.272, respectively. The mediating effect through FFs and SSBs was 0.033 and 0.010. Also, this mediation model has three mediation paths as follows: ST → FFs → Depressive symptoms, ST → SSBs → Depressive symptoms and ST → FFs → SSBs → Depressive symptoms. The ratio of the mediating effects of these three paths to the total mediating effect was 70.6, 5.9, and 23.5%, respectively, and the path coefficient tests were statistically significant (*p* < 0.001). Furthermore, sensitivity analysis shows that the estimated path coefficients are consistent in the structural equation model and the Bayesian model. However, the total mediating effect value increased by 0.001, and the path coefficient from ST to SSBs was not statistically significant.

**Table 3:** Mediating effects of FFs and SSBs in the association between ST and depressive symptoms in adolescents.

Effects	Paths	Bayesian model			Structural equation model		
		Estimate	*Posterior S.D.*	*95%* C.I.	Estimate	*S.E.*	*95%* C.I.
Direct	F1 → F4	0.125^**^	0.008	0.109–0.141	0.125^**^	0.009	0.107–0.142
	F2 → F4	0.112^**^	0.009	0.094–0.129	0.112^**^	0.009	0.094–0.129
	F3 → F4	0.092^**^	0.009	0.074–0.109	0.092^**^	0.010	0.072–0.110
	F1 → F2	0.219^**^	0.008	0.204–0.235	0.219^**^	0.009	0.202–0.238
	F1 → F3	0.018^*^	0.008	0.002–0.033	0.018	0.010	−0.001–0.036
	F2 → F3	0.419^**^	0.007	0.405–0.433	0.419^**^	0.012	0.397–0.442
Indirect	F1 → F2 → F4	0.024^**^	0.002	0.020–0.029	0.024^**^	0.002	0.020–0.029
	F1 → F3 → F4	0.002^**^	0.001	0.000–0.003	0.002^**^	0.001	0.000–0.003
	F1 → F2 →F3 →F4	0.008^**^	0.001	0.006–0.010	0.008^**^	0.001	0.007–0.010
	F1 → F4	0.034^**^	0.002	0.030–0.039	0.035^**^	0.003	0.030–0.039

**p < 0.001; *p < 0.05.

F1: Screen time; F2: Fast foods; F3: Sugar-sweetened beverages; F4: Depressive symptoms.

**Figure 1:**
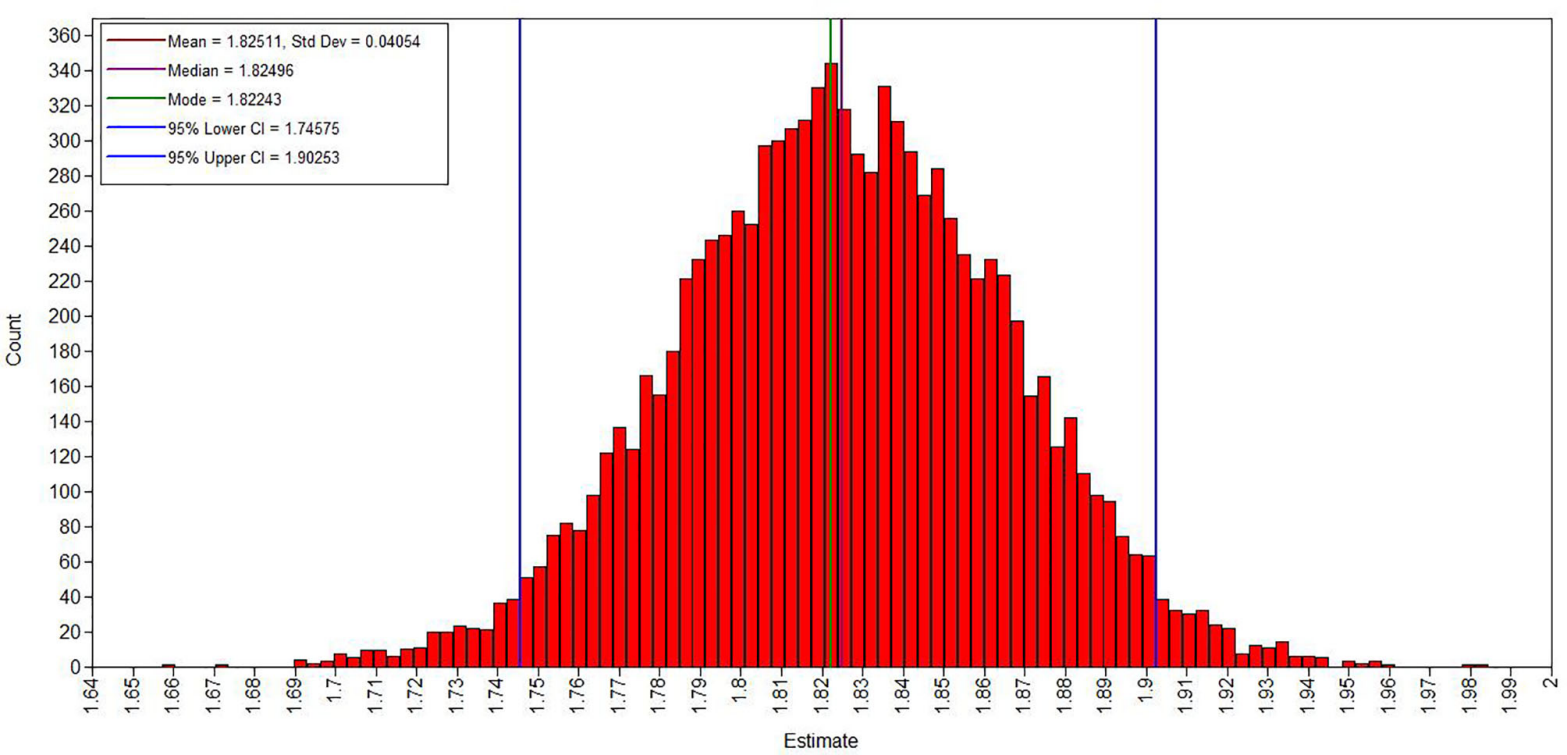
Bayesian posterior parameter distributions.

**Figure 2:**
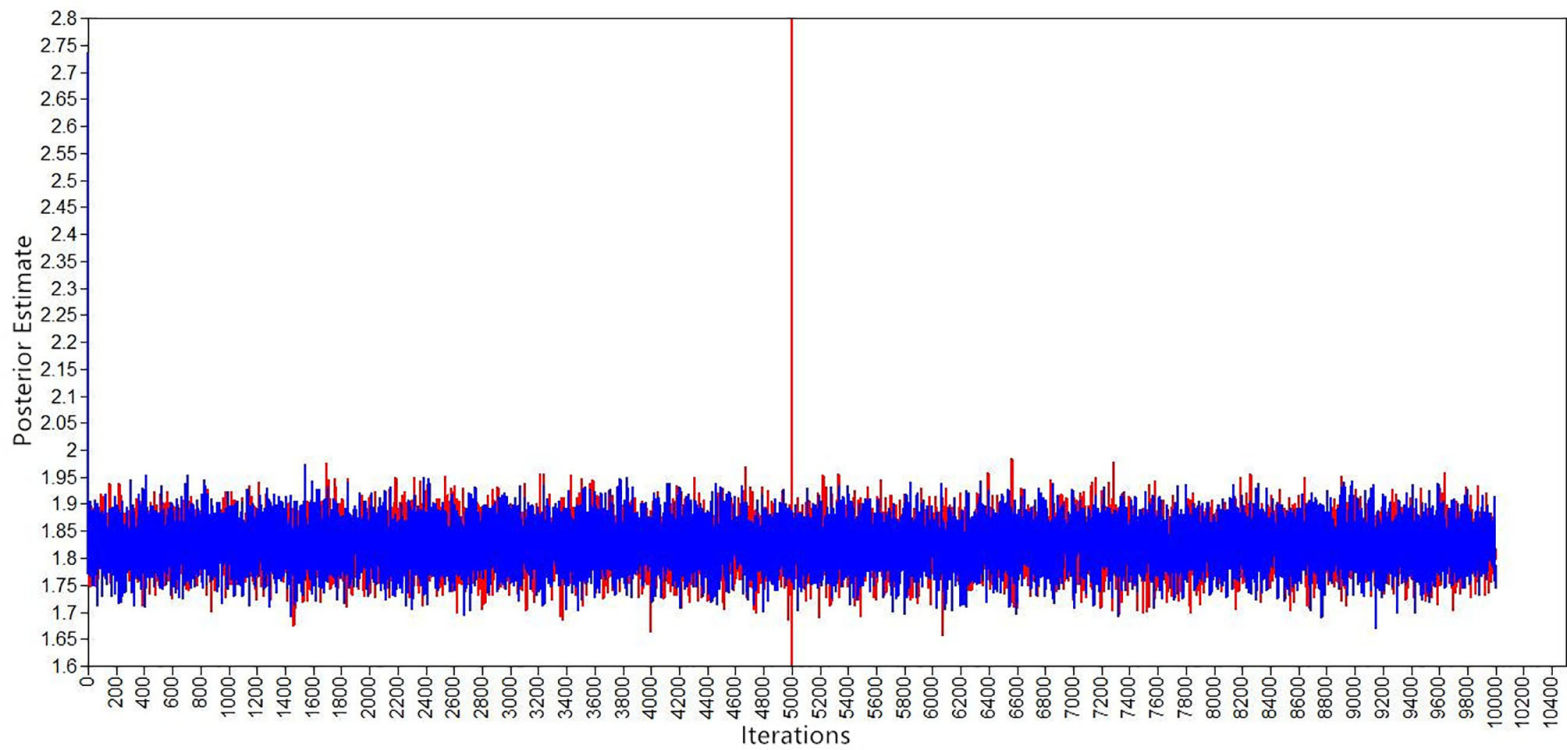
Bayesian posterior parameter trace plots.

**Figure 3:**
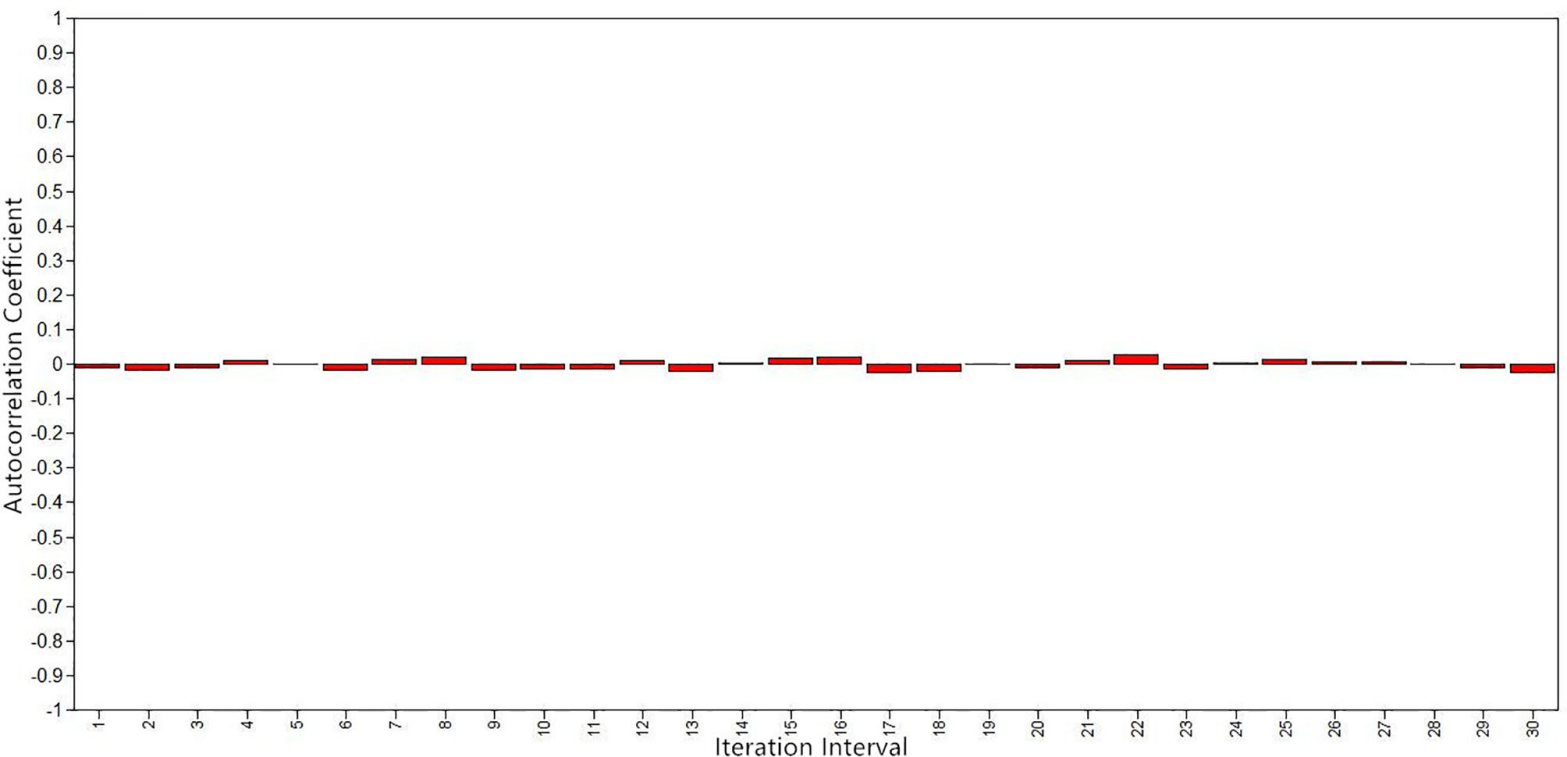
Bayesian autocorrelation plots.

**Figures 1** **–** **3** show the evaluation of parameter convergence in Bayesian model. **Figure 1** shows the Bayesian posterior parameter distributions. The posterior distribution is close to normal distribution in Bayesian posterior parameter distributions plots. **Figure 2** shows the Bayesian posterior parameter trace plots. The two Markov chains reached a steady state after 10,000 iterations in trace plots. **Figure 3** shows the Bayesian autocorrelation plots. The autocorrelation coefficient is less than 0.1 in Bayesian autocorrelation plots. Bayesian posterior parameter distributions plots, trace plots and Bayesian autocorrelation plots are used to evaluate the convergence of a model. Overall, Bayesian mediation model parameter convergence is reasonable.

**Figure 4:**
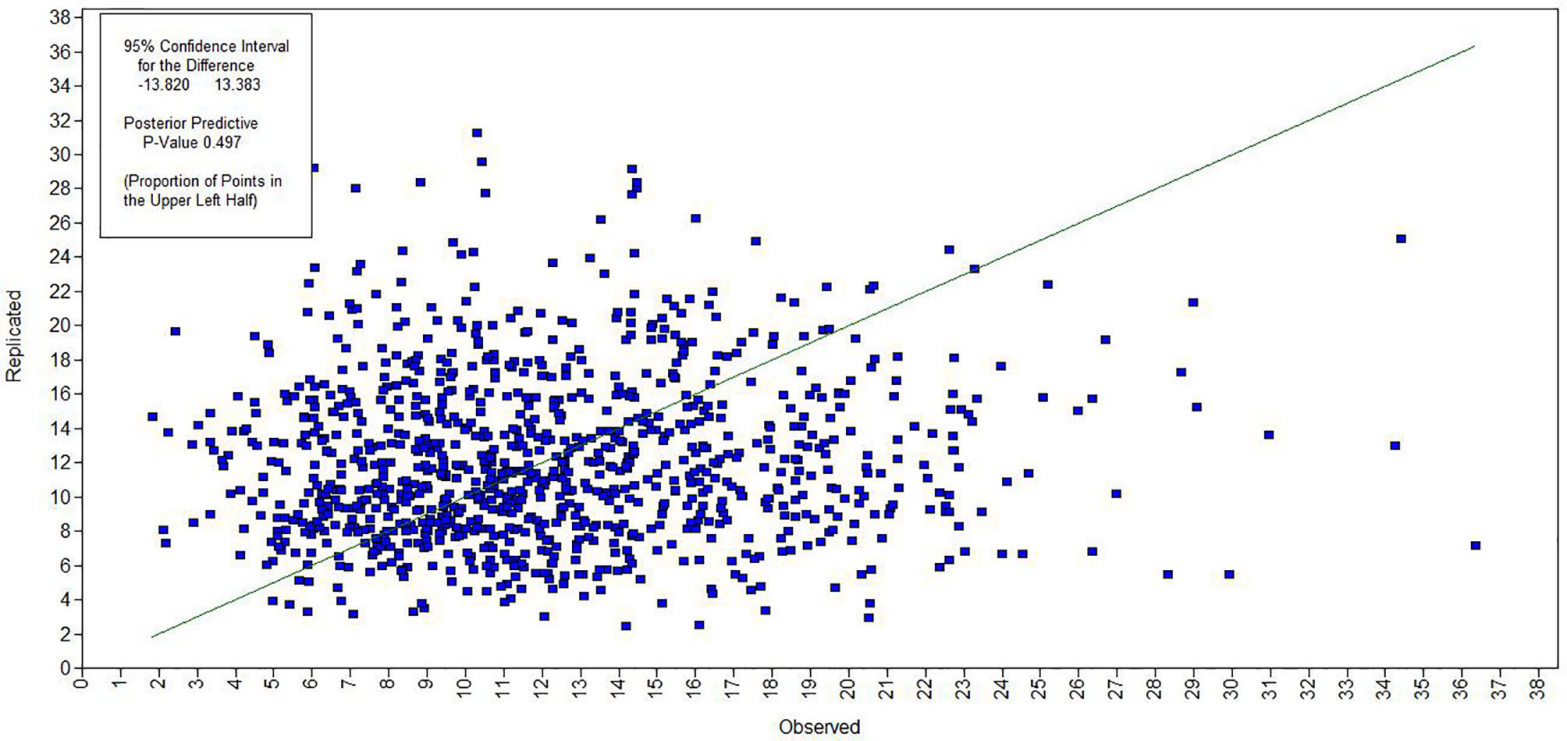
Bayesian posterior predictive checking scatter plots.

**Figure 5:**
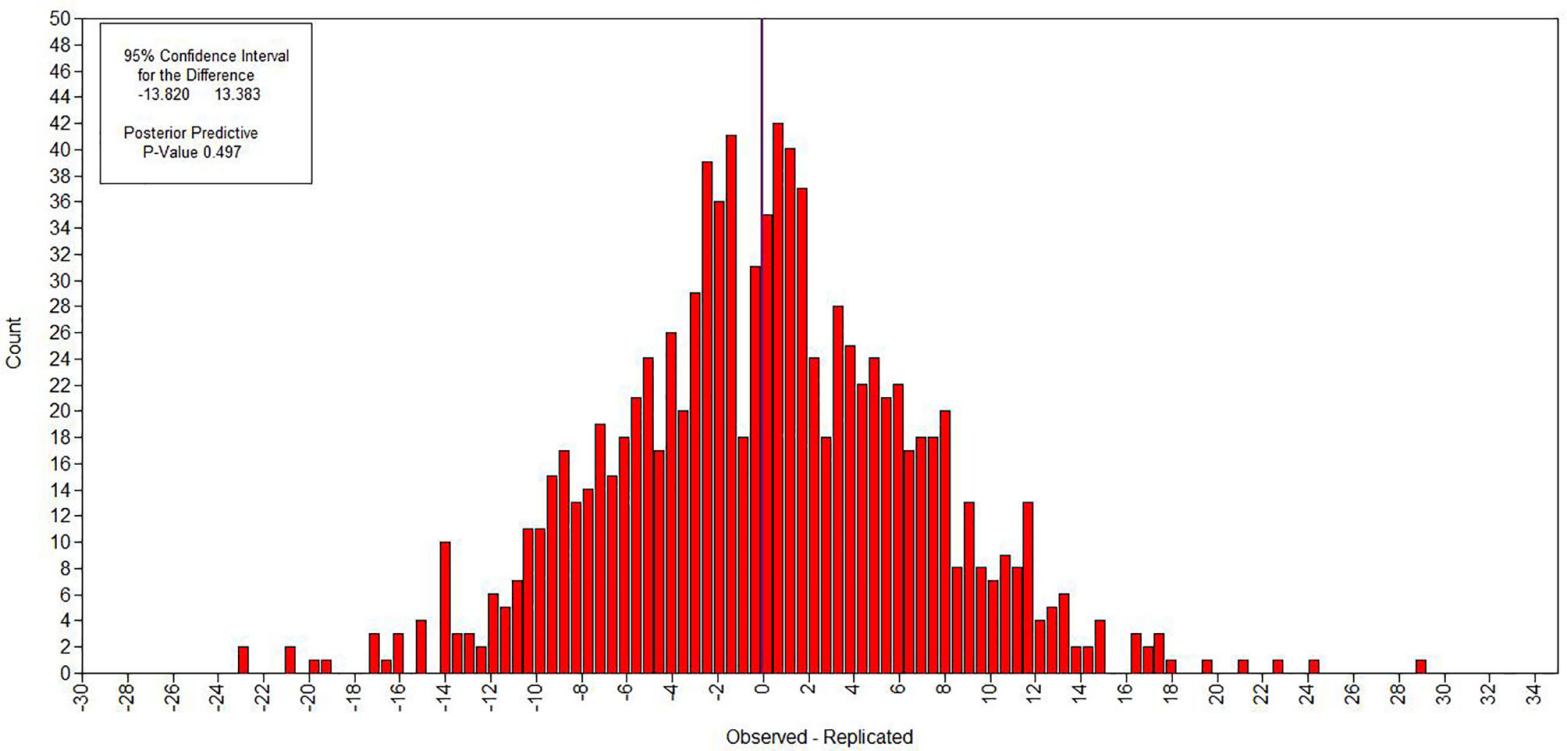
Bayesian posterior predictive checking distribution plots.

**Figures 4** and **5** show the evaluation of model fitting. A 95% confidence interval for the difference between the observed and the replicated chi-square values was (−13.820, 13.383), and posterior predictive p-value was 0.497, which is greater than 0.05. Additionally, Bayesian information criterion (BIC) was 228,842.421, and deviance information criterion (DIC) was 228,751.817. **Figure 4** shows the Bayesian posterior predictive checking scatter plots. Most of the scatters were observed fall on the 45^○^ line in the scatter plot. **Figure 5** shows the Bayesian posterior predictive checking distribution plots. The histogram is continuous and the observed data results are in the middle of the distribution in the histogram. The scatter plots and posterior predictive checking distribution plots are often used to evaluate the goodness of model fit. Thus, the results show that the model had a good fitting.

## Discussion

The positive rate of depressive symptoms in adolescents in our study was higher than that in developed countries such as Japan (22.0%) and France (12.6%) (7, 8), and higher than the results of research in developing countries such as Jamaica (14.2%) and Bangladesh (25%) (43, 44). It is notable that our results are lower than the findings (44.3%) of another study using CDI in Chinese adolescents (6). Additionally, our finding that there was no gender difference of positive rate of depressive symptoms, which was somewhat inconsistent with the previous study (44). In general, our results are compatible with previous findings indicating that the positive rate of depressive symptoms in Chinese adolescents is high, and adolescent depressive symptom needs paying close attention.

The results of logistic regression showed that observed stronger associations between the positive rate of depressive symptoms, ST, FFs and SSBs in the logistic regression model. These findings are consistent with previous studies (45, 46). Previous studies suggest that TV time is more than 2 h per day on weekdays related to the increased risk of depression in boys (*OR* = 1.33, 95% CI: 1.02–1.73) and girls (*OR* = 1.62, 95% CI: 1.19–2.21) (13). Other studies revealed that consumption of FFs and SSBs is associated with depressive symptoms in adolescents (44, 47).

There is an argument that frequent consumption of FFs and SSBs lead to a higher intake of energy, saturated fat and sugar, and lower intake of vitamins A and C, milk, fruits, and vegetables (48). However, the latter is inversely associated with adolescent depression (49). On the contrary, the former is positively associated with adolescent depression (50). Moreover, these findings are consistent with previous studies on FFs related to SSBs (48, 51), and ST related to FFs and SSBs (45).

Our observations imply that FFs and SSBs play a role of an intermediary factor in the association between ST and depressive symptoms, and 21.4% of the effects are mediated by FFs and SSBs. The mediating effect through FFs is about three times as large as that through SSBs. Likewise, from the results of sensitivity analysis, the path coefficient from ST to SSBs is not statistically significant in the structural equation model. Therefore, it is clear that FFs is the most important mediator in this mediation model compared to SSBs.

Mediating effect analysis can explore the internal mechanism of variable associations (52). On the basis of this theory, our study explored partial mediating mechanisms between ST and depressive symptoms in adolescents. 78.6% of direct effect indicates that ST is a pivotal predictor of adolescent depression. Namely, adolescents addicted to smartphones, internet, and television may have a higher probability of developing depressive symptoms. Also, the mediating effect of the path only by FFs accounts for the highest proportion of the total mediating effect (70.6%) among the three mediation paths observed in the mediation model. Nevertheless, the proportion of path only by SSBs was lower (5.9%). It should be noted that the chain mediation effect of FFs and SSBs accounted for 23.5% of the total mediating effect. Previous researches reported that FFs and SSBs consumption could promote each other (48), co-consumption was more significantly associated with depressive symptoms in adolescents (51, 53). Generally, the path (ST → FFs → Depressive symptoms) is the main mediation path associated with depression time in adolescents, and chain mediation path (ST → FFs → SSBs → Depressive symptoms) strengthened the association. Possible explanations for our findings are from the following three perspectives.

In terms of social behavior, first of all, adolescents eat FFs instead of irregularities in the diet because of being addicted to use smartphones, computers and TV for online games, surfing the internet, or TV programs for a long time. This is a common phenomenon in China. Second, FFs restaurants (e.g., KFC, Pizza Hut, etc.) can be seen everywhere in China with the progress of internationalization. Some Chinese adolescents are fond of eating Western-style FFs, often consume FFs and use smartphone take a long time to leave the fast food restaurant and even stay for a whole day. What’s more, there is an argument that long ST affects interpersonal and social functions, reduces communication with family and friends, even social isolation, which may lead to depressive symptoms (54). It is interesting to note that, there may be reverse associations, that is, depressed adolescents reduce interpersonal communication and long ST, and consume more FFs and SSBs.

In terms of neuropsychological and neuroimaging, the long ST and the frequent consumption of FFs and SSBs are closely related to the abnormal function of the brain’s reward circuits. The function of the prefrontal cortex, especially the execution control function, is associated with internet addiction (55). However, dysfunction of execution control network is highly correlated with uncontrolled eating behavior (56). Recent studies have indicated that people are willing to pay more for fat and carbohydrate and that this reward is associated with response in areas critical including the dorsal striatum and mediodorsal thalamus, which is related to greater recruitment of central reward circuits (57). Similarly, dependence behaviors, such as smartphone dependence and internet addiction, are associated with dysfunction of reward circuits, which can result in mental and behavior problems, including but not limited to maladjustment of pressure, reduced social response, and depression (58). In terms of molecular biology, firstly, FFs and SSBs account for a growing proportion of the diet in adolescents (48), and plastic packaging materials are used extensively could create exposure to bisphenol A (BPA) and phthalic acid ester (PAEs) (59, 60), and exposure to BPA and PAEs is associated with adolescent depressive symptoms (61, 62). Secondly, adolescents are the main consumers of SSBs that caused caffeine exposure (63); next, caffeine exposure is associated with depression (64–66). Thirdly, FFs contain high total fat, saturated fat and energy density, low micronutrient density, and a large amount of pro-inflammatory cytokines (67).

Several studies have suggested that pro-inflammatory diet such as FFs and SSBs are significantly associated with increased risk of depressive symptoms compared with anti-inflammatory diet (68, 69). Dietary inflammatory index is positively correlated with the risk of mental health problems (70). Adolescents who frequently consume FFs and SSBs may consume fewer fruits and vegetables. Vitamins, antioxidants, β-carotene and minerals in vegetables and fruits are associated with lower levels of inflammation and oxidative stress markers (71). A study of Australia adolescent aged 17 years suggests that Western dietary patterns were associated with body mass index (BMI) and inflammation biomarkers; and the association of Western dietary patterns and depression through biologically plausible pathways of adiposity and inflammation (72). Previous studies have shown that excessively long ST time of adolescents is associated with obesity and emotional symptoms such as depression and anxiety (11). Frequent consumption of SSBs and FFs were associated with adolescent obesity (73). Obesity is an important risk factor for mental problems such as inferiority, anxiety and depression (74). Furthermore, adolescents with long ST have relatively insufficient physical activity (75). Therefore, ST, FFs, SSBs, obesity and mental problems affect each other. Long-term unhealthy eating habits (e.g. watching TV, using computers and smart phone while consuming fast food and drinks) may cause obesity and affect the physical and mental development of adolescents (76).

Taken together, our data support the hypotheses that consumption of FFs and/or SSBs during ST may enhance the association with depressive symptoms in adolescents. Currently, the treatments could address one-third of the disease burden of depression (77). Therefore, prevention may be key to adolescent depression. Interventions could prove to be beneficial for preventing adolescent depressive symptoms. For instance, consumption of processed foods, FFs and SSBs should be limited, and the intake of anti-inflammatory diets (e.g., fruits, vegetables and nuts, etc.) should be increased in adolescents (77). Moreover, increasing physical activity time could be effective in reducing ST (78, 79). All that matters are avoiding consumption of FFs and SSBs, especially co-consumption, while using smartphones, computers, and watching TV.

Our study has several strengths. Above all, it’s a survey of the Chinese adolescent population, and our results can be transposed to other populations for public health and clinical practice due to the population-based setting. Moreover, this is the first study, to our knowledge, to find the mediation effect of FFs and SSBs in the association between ST and depressive symptoms in adolescents. Additionally, 14,500 adolescents were sampled and surveyed from 32 middle schools in four provinces in China, with a wide sampling range and a large sample size. Our study has several limitations that deserve discussion. Firstly, the causal association inference isn’t very strong because the data we used in our study were derived from cross-sectional surveys. There is a possible inverse association between screen time and depressive symptoms. Secondly, although our analysis shows that the mediation effect of ST in the association between FFs and SSBs consumption and depressive symptoms is not obvious, we still cannot explain clearly why FFs and SSBs lie on the pathway between ST and depressive symptoms. While there are some studies showing associations between ST, FFs, SSBs and depressive symptoms, there is no strong evidence presented why ST should lead to an increase in FFs and SSBs consumption. Further longitudinal studies are needed to clarify the associated direction and above questions. Thirdly, due to the general concern about adolescent mental health and discrimination against mental illness, adolescents are more sensitive to mental survey and may cover up their true mental conditions during the investigations. Therefore, there may be some social desirability bias in the research. However, we have taken some measures to reduce bias as much as possible. For instance, anonymous surveys establishing the good cooperative relations with schools, mobilizing adolescents to actively participate in the survey. Fourthly, this study used CDI to assess participants’ depressive symptoms and did not conduct clinical interviews. It is difficult to conduct clinical interviews to assess psychological symptoms in a large sample survey. CDI is widely used in the assessment of adolescents’ depressive symptoms with high reliability and validity, and the assessment results can reflect the mental health of adolescents. Fifthly, the consumption of FFs and SSBs in adolescents and ST are difficult to evaluate quantitatively. Consequently, we use consumption frequency to assess FFs and SSBs, and use subjective indicators to assess ST. For large sample association studies, the results should be meaningful. Sixthly, although the sample size of this study was large and the participants were distributed in four provinces of China, it may not be able to fully represent the Chinese adolescent population. From the geographical area of China, the sample of this study does not include North China, Northwest China and Northeast China. The eating habits of northern China may be different from other regions. However, this study focuses on the adolescent FFs and SSBs consumption behavior, not eating habits or dietary structure. The consumption of FFs and SSBs is a widespread phenomenon in Chinese adolescents, and there is little regional difference. In addition, obesity may be a factor that affects adolescents’ mental problems. However, this study did not measure adolescent body weight. We will further study the association of ST, FFs, SSBs, obesity and depressive symptoms in adolescents.

## Conclusion

Overall, we found that ST, FFs and SSBs consumption are associated with depressive symptoms in Chinese adolescents. FFs and SSBs consumption may play a role of mediating variable in the association between ST and depressive symptoms. There are chain-mediating effects. Thus, reducing ST and avoiding consuming FFs and SSBs during ST may be one of the effective measures to prevent adolescent depression.

## Data Availability Statement

The dataset supporting the conclusions of this article is available in the Mendeley Data repository, in http://dx.doi.org/10.17632/vy3wm76zzz.1. Further inquiries can be directed to the corresponding author.

## Ethics Statement

The principles of anonymity, confidentiality and voluntariness were strictly followed in our study. Participants and their guardians provided written informed consent, and students can withdraw from the study at any time. This study was approved by the Ethics Committee of Anhui Medical University (batch number: 20140087).

## Author Contributions

FT designed the study. HoX took primary responsibility for writing the manuscript, managed the literature searches and analyses, and undertook the statistical analysis. All other authors undertook the acquisition of the data, contributed to and have approved the final manuscript.

## Funding

This work was supported by the National Natural Science Foundation of China (81330068).

## Conflict of Interest

The authors declare that the research was conducted in the absence of any commercial or financial relationships that could be construed as a potential conflict of interest.
